# Modular Online Learning Design: A Flexible Approach for Diverse Learning Needs

**DOI:** 10.29173/jchla29535

**Published:** 2021-04-02

**Authors:** Kristy Hancock

**Affiliations:** Librarian Educator, Library Services, Nova Scotia Health, Yarmouth, Nova Scotia, Canada

Hess AN. **Modular Online Learning Design: A Flexible Approach for Diverse Learning Needs**. 1st ed. Chicago, IL: ALA Editions; 2021. Softcover: 144 p. ISBN: 978-0-8389-4812-5. Price: USD $65.99. Available from: https://www.alastore.ala.org/content/modular-online-learning-design-flexible-approach-diverse-learning-needs

With COVID-19 limiting in-person information literacy programming across Canada, support for online library instruction is timely. In *Modular Online Learning Design: A Flexible Approach for Diverse Learning Needs*, author Amanda Nichols Hess presents a modular approach to creating online learning resources in libraries. Hess refers to modularity as the ability for a learning resource to function as a freestanding object or in combination with other learning resources. The central idea of modular online learning design is to create flexible content that can easily be reused, updated and adapted for different contexts and to meet different learning needs. According to Hess, modularity can be achieved by applying a forward-thinking approach when creating, maintaining, updating and evaluating learning resources.

Hess is more than qualified to provide guidance on the topic of online learning design. She has conducted extensive research on information literacy instruction and online learning, among other topics, and has experience creating online learning resources in school and academic libraries. She is an associate professor and the coordinator of instruction and research help at Oakland University in Rochester, Michigan.

Even though the author’s focus is on instruction in academic libraries, *Modular Online Learning Design* is relevant to library staff in any setting who create learning objects such as video tutorials, self-paced modules, instructional handouts and subject guides. The example projects used and web resources suggested in the text are specific to academic libraries, but the structure of the design process and the planning tools are applicable to special, public and school libraries as well. While there is no shortage of resources for librarians about designing effective learning resources, this is the first book about online learning design that I have read.

Laid out in nine short chapters, *Modular Online Learning Design* is organized logically, echoing a realistic approach to the design process. The book covers topics such as selecting an appropriate instructional design approach; strategies for aligning projects with program, organizational and professional goals; getting buy-in, participation and support from stakeholders; and identifying, using and adapting existing online learning content. Hess goes on to discuss accessibility in online learning environments and the principles of Universal Design for Learning; measuring the impact of learning objects through testing, evaluation and assessment; and scaling resources to employ them for a range of needs and audiences. In the final chapter Hess focuses on capacity and procedure requirements for maintaining content in the long term to ensure its continued relevance. Although valuable to read front to back, a highlight of this work is that readers can dive in at any chapter, since they do not need to be read sequentially.

While the book is a useful introduction to modular online learning design, Hess neglects to provide enough detail about certain aspects of the design process. For example, copyright and Creative Commons (CC) licensing are only briefly mentioned in chapter 5, “Modifying and Adapting Existing Content,” and chapter 9, “Forward Thinking for Future Modularity.” In chapter 5, an overview of the different CC license types as well as copyright considerations for reusing content without a CC license would be helpful. In chapter 9, guidance about selecting a CC license for one’s own learning objects would be a valuable addition. Prospective readers should be aware that they will likely need to consult additional in-depth resources for practical guidance during some stages of the design process. In this way, *Modular Online Learning Design* falls short of being as comprehensive a guide as it could have been.

Some aspects of the book are more practical. In each chapter, the concepts introduced are applied in example projects of different scopes so as to demonstrate how librarians can put the concepts into practice. Planning tools such as checklists and charts are peppered throughout the book, and they can be used as is or adapted to fit the specific needs of an online learning initiative. In chapter 6, “Designing for Accessibility and Equitable Experiences,” Hess provides tangible guidance related to web accessibility in online learning environments, and in chapter 5, “Modifying and Adapting Existing Content,” Hess suggests some open educational resource repositories for finding and sharing information literacy materials.

Even though there are some gaps in coverage, I would recommend *Modular Online Learning Design* to library staff in any setting who create learning objects and who are new to the idea of modular online learning design. Realistically, due to time constraints, librarians looking to take direction from Hess’ approach may need to prioritize certain stages of the design process, depending on the project scope and objectives. Those with extensive experience creating content for online learning environments may not find the content novel or useful. The book is more of a starting point rather than a comprehensive guide, but because of its positive aspects, I think *Modular Online Learning Design* represents good value for the price. As a health sciences librarian in a hospital, I intend to apply some of the concepts and planning tools to the online learning resources that I create.

